# The Factory System of the United States

**Published:** 1893-04-15

**Authors:** 


### THE PRACTITIONERS' BOOKSHELF.

### THE FACTORY SYSTEM OF THE UNITED STATES.*

We are so accustomed in England to look on the factory system as one of the great institutions of this country necessarily associated with certain evils which constantly require the services of Parliament to repress, that any publication attempting to prove that the advantages outweigh the disadvantages will receive the careful attention of those interested in the health and welfare of our factory hands. Though written nearly ten years ago by an American for the Department of the Interior of the United States, the materials which Mr. Carroll Wright has collected are borrowed largely from European sources, and even if they were not so, the report would be full of valuable and interesting information as containing a careful and concise statement on the factory system of America.

Mr. Wright commences his report with a brief but very interesting history of the cotton industry, taking that manufacture as an example, and shows how the honour of inventing the spinning machine was due not to Arkwright, as is generally supposed, but to Wyatt, who patented a machine thirty years previously. It was, perhaps, a re-discovery of Arkwright's, but, at any rate, to him is due the honour of making popular the spinning machine, though it was by the assistance of John Smalley, the liquor merchant, who supplied the money. After these came the inventions of Hargreaves and Crompton, and, finally, the steam engine of Watt. By these inventions the labourer was taken from his home to work in the factory, resulting, as Mr. Wright believes, in his moral and physical improvement. In spite of legislation and the threat of heavy penalties, it was found impossible to keep the inventions in England, and in a few years mills and factories had sprung up on the other side of the Atlantic, but even to the present day the number is small compared with that of England, for one Lancashire town alone—Oldham—possesses more spindles in motion than the whole United States. Mr. Wright then, in a few words, compares the advantages of the factory system over the older one, which he called the "domestic," showing how the gradual disappearance of the hand machines was coincident with an improvement in the moral, social, and physical conditions of the labourer.

Whilst admitting that many serious evils exist in factory towns, Mr. Wright believes that the mill is not the cause of the evils, but that though these evils exist, as he admits, they do not necessarily arise from the system. That the factory is responsible, as many of its opponents say, for intemperance, unthrift, poverty, prostitution, and intellectual degeneration, Mr. Wright unhesitatingly denies, and brings forward as many statistics as are possible to collect to show that the factory hand is temperate, economical, and daily improving in mental power, while the factory girl is modest and virtuous, and far better off than the milk-maid. One evil Mr. Wright admits exists, and this is that the work of mothers is detrimental to the life of the unborn and living children. In Germany, and in other places, allowances are made to expecting mothers, and we believe, from our experience in manufacturing villages in Yorkshire, that it is far from common for a woman far advanced in pregnancy to go to the mill.

Mr. Wright's statistics and quotations on the influence of

* "Report on the Factory System of the United States." By Carroll D. Wright. 78 p.p. (Government Printing Office, Washington, 1884.)

the factory system on wages, prices, and production we regret, on account of space, we are unable to review at any length, but they all tend to prove that the workman is far better off than he was in the time of the "domestic" system. Factory legislation, a subject which has received so much attention of late years, is traced from the Apprentice Act of Elizabeth (1562) to the present date (1884), showing how Sir Robert Peel, himself a large mill owner, and impressed with the evils that had arisen by the employment of pauper children, introduced the first Factory Act. Brief *resumés* of the latest Acts in force in England, Germany, France, and America are given, but Mr. Wright calls attention to the different circumstances under which factory labour exists in the various countries mentioned, for whereas in Europe each country to a large extent supplies its own labour, the operative class of America consists of people drawn from all quarters of the globe. Specimens, with illustrations, of the model homes of workmen are given, and a few words are said about the tenements and flats common in parts of England, and the report closes with a few remark on the future of the factory system.

From first to last we have read Mr. Carroll Wright's report with great interest, and we heartily recommend it to those who are interested in the conditions of the working classes.

